# Intracoronary Glycoprotein IIb/IIIa Inhibitors Improve Short-Term Mortality and Reinfarction in East Asian Patients with ST-Segment Elevation Myocardial Infarction after Thrombus Aspiration: A Meta-Analysis

**DOI:** 10.1155/2018/5174714

**Published:** 2018-08-15

**Authors:** Jia-hong Wu, Pan-pan Hao, Yu-guo Chen, Rui-jian Li

**Affiliations:** ^1^Department of Radiology, Qilu Hospital of Shandong University, Jinan 250012, China; ^2^Department of Cardiology, Qilu Hospital of Shandong University, Jinan 250012, China; ^3^Department of Emergency, Qilu Hospital of Shandong University, Jinan 250012, China

## Abstract

**Objective:**

Intracoronary (IC) glycoprotein IIb/IIIa inhibitors (GPIs) after thrombus aspiration (TA) for patients with ST-segment elevation myocardial infarction (STEMI), as compared with percutaneous coronary interventions (PCI) alone, is still on debate. To address this issue, we performed a meta-analysis of results from prospective or randomized controlled trials on the topic.

**Methods:**

We searched electronic and printed sources (up to June 20, 2016) according to the selection criteria. Data were abstraction and meta-analysis was performed using RevMan 5.3 software.

**Results:**

The cohorts involved 14 articles describing 1,918 participants were included. The incidence of the short-term major adverse cardiac events (MACE) was significantly reduced with intracoronary GPIs after TA (odds ratio [OR]: 0.29; 95% confidence interval [CI]: 0.13 to 0.65, p=0.003). Benefits were noted for short-term mortality (OR: 0.31; 95% CI: 0.17 to 0.57, p=0.0002) and reinfarction (OR: 0.28; 95% CI: 0.10 to 0.78, p=0.01) in subjects who received intracoronary GPIs after TA. Moreover, the Thrombolysis in Myocardial Infarction (TIMI) trial grade 3 postprocedure (OR: 2.29; 95% CI: 1.72 to 3.04, P<0.00001) and complete ST-segment resolution (STR) rate (OR: 2.68; 95% CI: 1.85 to 3.87, P<0.00001) were both improved with intracoronary GPIs after TA. As a result, left ventricular ejection fraction (LVEF) at short-term follow-up showed a significant difference (OR: 7.33; 95% CI: 5.60 to 9.06, p<0.0001) in favor of the TA and intracoronary GPIs administration.

**Conclusions:**

Our study demonstrates that intracoronary GPIs may have a synergistic effect with thrombus aspiration on short-term mortality, reinfarction, and cardiac functional recovery.

## 1. Introduction

Primary percutaneous coronary intervention (PCI) has become the preferred reperfusion modality for patients with acute ST-segment elevation myocardial infarction (STEMI) [1]. As we all know, the possibility of distal embolization of atherosclerotic plaque and thrombus with subsequent microvascular injury and increased infarct size during primary PCI is associated with adverse cardiovascular events [2]. Thrombus aspiration (TA) has the potential of reducing distal embolization and improving microvascular perfusion during primary PCI. Even though numerous international studies have been reported, there are still conflicting results on the clinical impact of thrombus aspiration during primary PCI [3, 4]. Recent evidence from* Routine Aspiration Thrombectomy With Percutaneous Coronary Intervention (PCI) Versus PCI Alone in Patients With ST-Segment Elevation Myocardial Infarction (STEMI) Undergoing Primary PCI (TOTAL) trial*, the largest trial of thrombus aspiration in STEMI so far, suggested that routine thrombus aspiration, as compared with PCI alone, did not reduce the risk of major adverse cardiovascular events (MACE) within 180 days [5], consistent with those of* Thrombus Aspiration during ST-Segment Elevation Myocardial Infarction (TASTE) trial* [4] and* the Intracoronary Abciximab and Aspiration Thrombectomy in Patients With Large Anterior Myocardial Infarction (INFUSE-AMI) trial* [6]. However, TA along with intracoronary (IC) glycoprotein IIb/IIIa inhibitors (GPIs) was associated with improved 30-day mortality in INFUSE-AMI trial [6], which suggested the synergistic effect of TA and GPIs might be attributed to improvement in clinical outcomes. On the other hand, some East Asian studies (especially in China) from the year 2008 to 2015 yielded conflicting or inconclusive results [6, 7]. The reason for the discrepancy is unclear but may be related to low statistical power or difference among the ethnic groups studied.

In this meta-analysis, we aim to assess the effects of intracoronary GPIs after thrombus aspiration compared with PCI alone in STEMI patients from the year 2008 to 2015.

## 2. Methods

### 2.1. Data Sources and Searches

We performed a systematic search for articles in the databases MEDLINE (via PubMed), EMBASE, and the Cochrane Library (Cochrane Central Register of Controlled Trials) up to June 20, 2016, using the following keywords: (thrombus aspiration) AND (intracoronary) AND {(“abciximab”[Substance] or “abciximab”[All Fields]) or (“eptifibatide”[Substance] or “eptifibatide”[All Fields]) or (“tirofiban”[Substance] or “tirofiban”[All Fields]}. We also searched the China National Knowledge Internet database to retrieve relevant studies published in Chinese. We restricted the search to human studies but not language. Further articles were retrieved by a manual search of references from recent reviews and relevant published original studies. Studies were screened by reading the abstracts and titles and then selected after reading the full text.

### 2.2. Study Selection

A study was selected if (1) the subjects were prospectively or randomly assigned to TA plus GPIs or PCI alone in a parallel-group design; (2) major adverse cardiac events were reported as outcomes; (3) GPIs were administrated by intracoronary during the procedure. We excluded studies that were cross-sectional or case-control designs. In case of duplicate publication, we chose the publication reporting on the primary analysis. The long-term clinical outcome was defined as more than three months, and the short-term clinical outcome was less than three months or in hospital.

### 2.3. Data Extraction and Quality Assessment

Data were extracted independently by 2 investigators (Li R.J. and Hao P.P.) using a standardized extraction form and compared. Discrepancies were resolved by discussion with a third investigator (Chen Y.G.) and by referencing the original report. The grade of study quality was assessed as the previous meta-analysis [8, 9].

### 2.4. Data Analysis

RevMan 5.3, developed by the Cochrane Collaboration (http://tech.cochrane.org/revman, released on 13 June 2014), was used for the meta-analysis. Heterogeneity was tested with the chi-square and* I*^*2*^ tests. Statistical significance was a 2-tailed* P* < 0.05. Results showing no significant differences were analyzed by the fixed effects model and those showing significant differences were analyzed by the DerSimonian-Laird random effect model. We also performed a sensitivity analysis to explore the robustness of our results. For MACE, mortality, and reinfarction, we evaluated publication bias using funnel plots and the fail-safe number with* P* < 0.05 (Nfs0.05), Nfs0.05 = (ΣZ/1.64)^2^  − k, where k is the number of studies included in the meta-analysis. Any calculated Nfs value smaller than the number of observed studies indicated publication bias that might influence the meta-analysis results.

## 3. Results

**Figure 1:**
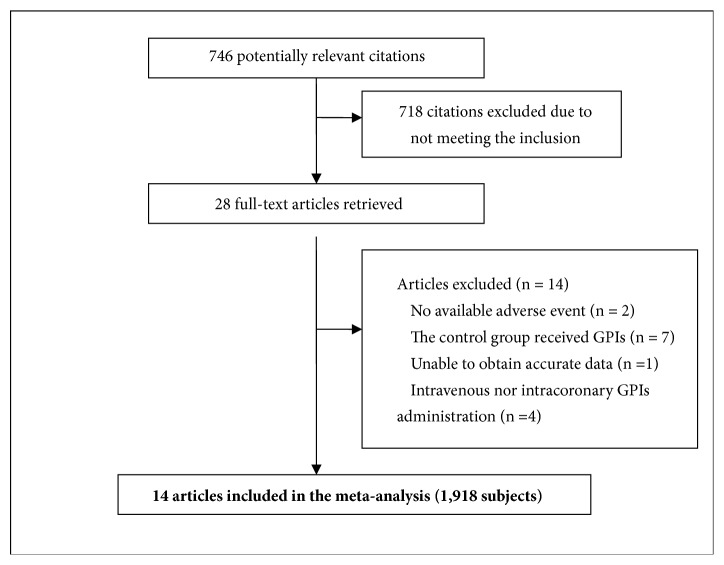
Selection of articles for the meta-analysis. GPIs: glycoprotein IIb/IIIa inhibitors.

A total of 14 observational studies with 1,918 participants were finally included (Figure 1) [7, 10–22]. The geographical distribution of 14 studies was all in East Asian regions. One study was from the Republic of Korea, 1 from Taiwan region, and the others from the mainland of China. Of 14 articles, 11 studies [10–20] reported the short-term clinical outcome; other 3 studies [7, 21, 22] reported the long-term clinical outcome (18 months, 12 months, and 6 months, respectively). Figure 1 reported study selection procedure, while Table 1 summarizes the most relevant characteristics of the selected studies.

Seven studies depicting baseline characteristics were summarized in Table 2. For these studies, baseline characteristics were not significantly different between the two groups. Choi's study was an abstract from ANGIOPLASTY SUMMIT in 2009 [22], and we could not obtain accurate baseline characteristics although we have contacted the corresponding author. Other 6 studies described those baseline characteristics were balanced between two groups in papers, while no accurate data of baseline characteristics were obtained from [15–20].

**Figure 2:**
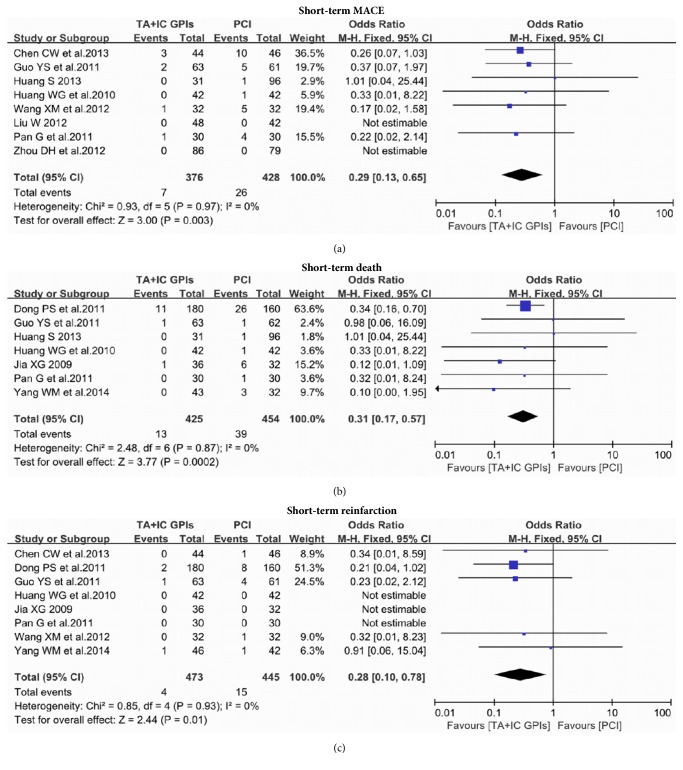
(a) The meta-analysis of MACE at the short-term follow-up; (b) the meta-analysis of death at the short-term follow-up; (c) the meta-analysis of reinfarction at the short-term follow-up. MACE: major adverse cardiac events; TA: thrombus aspiration; IC: intracoronary; GPIs: glycoprotein IIb/IIIa inhibitors; PCI: percutaneous coronary interventions.

There were 8 studies which reported short-term MACE after the procedure [10–12, 14, 16–18, 20]. The analysis for the short-term MACE revealed that the incidence of MACE was significantly lower in the patients treated with intracoronary GPIs after TA than those with PCI alone (1.86% versus 6.06%; odds ratio (OR): 0.29; 95% confidence interval (CI): 0.13 to 0.65, p=0.003; Figure 2(a)). In terms of short-term mortality, 7 studies reported the results [11–13, 15, 16, 18, 19]. The incidence of short-term mortality was significantly reduced in subjects who received TA and IC GPIs treatment (3.06% versus 8.59%; OR: 0.31; 95% CI: 0.17 to 0.57, p=0.0002; Figure 2(b)). Eight studies reported the short-term reinfarction rates [10, 12–16, 18, 19]. An obviously decreased risk of short-term reinfarction was observed in the TA and IC GPIs group compared with the PCI group (0.85% versus 3.37%; OR: 0.28; 95% CI: 0.10 to 0.78, p=0.01; Figure 2(c)).

**Figure 3:**
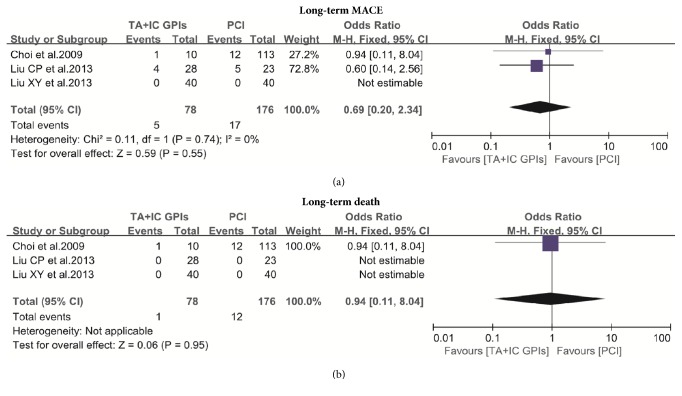
(a) The meta-analysis of MACE at the long-term follow-up; (b) the meta-analysis of death at the long-term follow-up. MACE: major adverse cardiac events; TA: thrombus aspiration; IC: intracoronary; GPIs: glycoprotein IIb/IIIa inhibitors; PCI: percutaneous coronary interventions.

Looking at the long-term MACE reported by 3 studies, our analysis did not show a significant difference between the two groups (6.41% versus 9.65%; OR: 0.69; 95% CI: 0.20 to 2.34, p=0.55; Figure 3(a)) [7, 21, 22]. Similarly, no significant difference was noted in long-term mortality between the two groups (1.28% versus 6.81%; OR: 0.94; 95% CI: 0.11 to 8.04; p=0.95; Figure 3(b)) [7, 21, 22]. The analysis of long-term reinfarction rate was unable to be performed due to only two studies reporting [7, 21].

**Figure 4:**
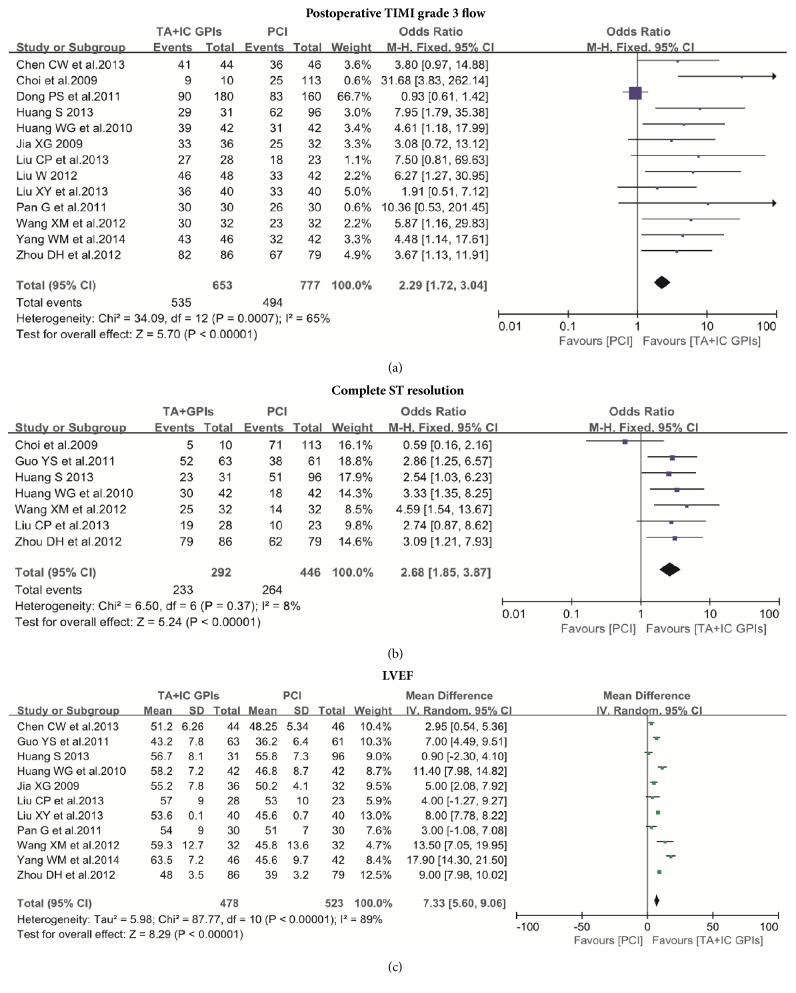
(a) Meta-analysis of postoperative TIMI grade 3 flow between thrombus aspiration plus intracoronary GPIs and PCI alone; (b) meta-analysis of postoperative complete ST resolution (STR) between thrombus aspiration plus intracoronary GPIs and PCI alone; (c) meta-analysis of LVEF before discharge or at the short-term follow-up between thrombus aspiration plus intracoronary GPIs and PCI alone. TIMI: the Thrombolysis in Myocardial Infarction trial; LVEF: left ventricular ejection fraction; TA: thrombus aspiration; IC: intracoronary; GPIs: glycoprotein IIb/IIIa inhibitors; PCI: percutaneous coronary interventions.

Thirteen studies reported the postprocedural flow grades based on the Thrombolysis in Myocardial Infarction (TIMI) trial [7, 10–15, 17–22]. The incidence of postprocedural TIMI flow grades 3 was higher in patients treated with TA and IC GPIs compared with those who did not (81.9% versus 63.6%; OR: 2.29; 95% CI: 1.72 to 3.04, P<0.00001; Figure 4(a)). Seven studies reported complete ST-segment resolution (STR) rate at 60 minutes~90 minutes after the procedure [7, 11, 12, 14, 16, 20, 22]. The incidence of postprocedural complete STR significantly increased in patients treated with TA and IC GPIs (79.8% versus 59.2%;OR: 2.68; 95% CI: 1.85 to 3.87, P<0.00001; Figure 4(b)). Subgroup analysis, according to TA catheter, showed that both postprocedural TIMI flow 3 and MACE were improved in studies using ZEEK aspiration catheter (Zeon Medical Inc., Tokyo, Japan) and EXPORT aspiration catheter (Medtronic, Minneapolis, Minnesota), whereas no benefit of MACE was observed in studies using Driver C.E. aspiration catheter (Invatec, Brescia, Italy) (Table 3).

**Figure 5:**
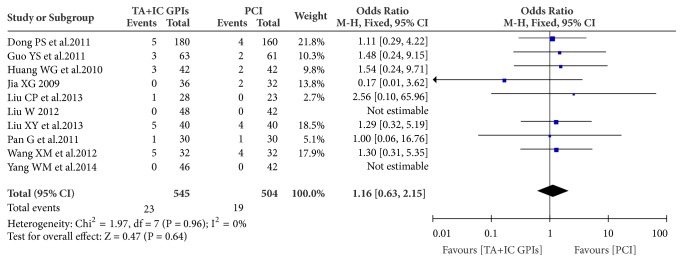
Meta-analysis of the major bleeding or minor bleeding events before discharge between thrombus aspiration plus intracoronary GPIs and PCI alone. TA: thrombus aspiration; IC: intracoronary; GPIs: glycoprotein IIb/IIIa inhibitors; PCI: percutaneous coronary interventions.

Importantly, the analysis of left ventricular ejection fraction (LVEF) before discharge or at short-term follow-up (reported by 11 studies) showed a significant difference (54.5% versus 47.0%; OR: 7.33; 95% CI: 5.60 to 9.06, p<0.0001) in favor of the TA and IC GPIs administration route (Figure 4(c)) [7, 10–14, 16, 18–21]. There were no significant differences in the major bleeding and minor bleeding events between the two groups (4.22% versus 3.77%; OR: 1.16; 95% CI: 0.63 to 2.15, p=0.64; Figure 5) [7, 12–19, 21].

**Figure 6:**
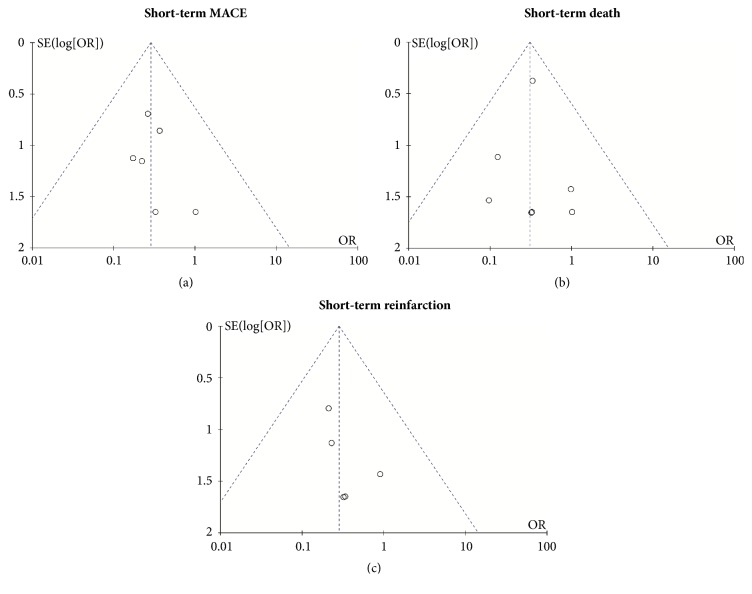
Funnel plot of publication bias for the short-term MACE (a), death (b), and reinfarction (c). MACE: major adverse cardiac events.

In heterogeneity testing and sensitivity analysis, we also found no significant heterogeneity for studies reporting short-term MACE, death, and reinfarction, and exclusion of any single study did not alter the overall finding. The funnel plot assessing the publication bias is shown in Figure 6. We calculated the Nfs0.05 for MACE, death, and reinfarction. The comparative Nfs0.05 for MACE, death, and recurrent MI of the short term was 10.29, 14.93, and 2.97, whereas those of long term were -0.99, -0.99, and -0.82, which indicated publication bias that might influence the meta-analysis results.

## 4. Discussion

The main findings of the present meta-analysis are as follows: (1) a combination of thrombus aspiration and intracoronary GPIs seemed to be superior to PCI alone in terms of enhancing myocardial perfusion, as assessed by postprocedural TIMI flow 3, and complete STR rate. Importantly, cardiac function at short-term follow-up, analyzed by LVEF, showed much better to be in the thrombus aspiration and intracoronary GPIs group over the PCI group. (2) The incidence of short-term MACE was significantly reduced with intracoronary GPIs after thrombus aspiration, including death and reinfarction, whereas there was no trend towards better outcome in studies with long-term MACE.

Thrombus aspiration during primary PCI is controversial, especially after the TOTAL trial which showed that routine thrombus aspiration did not reduce the risk of long-term MACE, as compared with PCI alone, and the findings are consistent with those of the INFUSE-AMI trial. However, a subgroup analysis of the INFUSE-AMI trial showed that thrombus aspiration plus intracoronary administration of GPIs improved myocardium perfusion and resulted in a better clinical prognosis [6]. A recent meta-analysis, summarizing the conflicting randomized controlled trials (RCTs) of comparing thrombus aspiration with the control arm, showed that thrombus aspiration along with GPIs is associated with improved 30-day mortality [23]. However, the authors performed metaregression and compared the studies with a higher proportion of glycoprotein IIb/IIIa inhibitor use and those with lower glycoprotein IIb/IIIa inhibitor use in the thrombus aspiration arm [23]. In contrast, we pooled the studies which directly compared thrombus aspiration plus GPIs with PCI alone, especially intracoronary administration. Our meta-analysis could demonstrate a reduced incidence of short-term MACE in the intracoronary GPIs after thrombus aspiration group, as compared to the PCI group. Further analysis showed that the benefit comes from reduced short-term mortality and reinfarction. Nonetheless, it has to be underlined that no benefit of long-term MACE was observed.

Ahn et al. found that distal embolization was less likely to occur in patients undergoing intracoronary abciximab and thrombus aspiration as assessed by on-site measurement of the index of microcirculatory resistance (IMR) after primary PCI [24], which suggested that combination treatment using GPIs and thrombus aspiration may synergistically improve myocardial perfusion in patients with STEMI undergoing primary PCI. It was suggested that direct intracoronary injection of GPIs might be superior to its intravenous injection for improving myocardial perfusion due to creating a higher local concentration of around the coronary thrombus, and high local concentration may facilitate thrombus disaggregation and improve microvascular perfusion [24, 25]. Our meta-analysis found that intracoronary GPIs after thrombus aspiration provided significant benefits in postprocedural TIMI flow grade 3, complete STR when compared with PCI alone. STR is a simple and reliable marker of effective myocardial reperfusion which correlates with cardiac functional recovery [26]. In the present meta-analysis, complete STR tends to be better in the intracoronary GPIs after thrombus aspiration group than that in the PCI alone group. This result coincided with the improvement in left ventricular function measured by LVEF and resulted in a trend towards better clinical outcomes.

A subgroup analysis of INFUSE-AMI [6] and a meta-analysis [27] suggest that a combination of thrombus aspiration and GPIs treatment is effective in decreasing infarct size and mortality as compared to each treatment alone or PCI alone. These findings are consistent with those of our meta-analysis. If most thrombotic materials are retrieved by thrombus aspiration catheter, GPIs could further dissolve residual thrombus and microemboli in the microvasculature. This might interpret why our results of the meta-analysis were different from other studies [4, 5]. Notably, subgroup analysis showed that the type of aspiration catheter might influence the clinical outcomes and, in this aspect, ZEEK catheter and EXPORT catheter, which present a stronger aspiration capacity for moderate to large thrombi, were superior to Driver C.E. catheter [28]. Differences in aspiration capacity between ZEEK, EXPORT, and Diver C.E. in this setting might influence the short-term outcome.

We found there was no significant difference between the number of bleeding events in the intracoronary GPIs group and those in the control group, despite GPIs' antiplatelet activity and the risk of bleeding. This might be due to the type of GPIs–tirofiban, which was mostly used in our meta-analysis (92.9% used tirofiban). Tirofiban is a representative of small molecule glycoprotein IIb/IIIa inhibitor with reliable platelet inhibition and reversibility [29]. Tirofiban, the most applied glycoprotein IIb/IIIa inhibitor in East Asia now, was found in 13 included studies (Choi's study not mentioned due to the abstract in ANGIOPLASTY SUMMIT).

There were also some limitations in this meta-analysis. First, few studies were found in other countries of East Asia, and we could not perform analyses in other populations. Second, we calculated the Nfs0.05 to assess the publication bias and found the results of long-term MACE, death, and reinfarction were -0.99, -0.99, and -0.82, which indicated publication bias and might influence the meta-analysis results. Third, 92.9% studies in our meta-analysis used tirofiban, and subgroup analysis of different type of GPIs was unable to be performed.

## 5. Conclusions

Our study demonstrates that intracoronary use of glycoprotein IIb/IIIa inhibitors may have a synergistic effect with thrombus aspiration on short-term mortality, reinfarction, and cardiac functional recovery. Future RCTs are needed to assess the impact of concomitant glycoprotein IIb/IIIa inhibitors with thrombus aspiration on the long-term outcomes of patients with STEMI.

## Figures and Tables

**Table 1:** Characteristics of the trials included in the meta-analysis.

Study	Year	Location	Study design	Patients included, PPCI or not	MACE	Mortality reported	Follow up (months)	TA catheter	Study quality
Choi	2009	Republic of Korea	Retrospective	STEMI,PPCI	mortality	Yes	18	Export	Fair
Liu XY	2013	Mainland China	Randomized	STEMI,PPCI	death, reinfarction, TLR	Yes	12	ZEEK	Good
Liu CP	2013	Taiwan	Randomized	STEMI,non-PPCI	death, reinfarction, TLR, and stroke	Yes	6	Thrombuster II	Good
Chen CW	2013	Mainland China	Randomized	STEMI,PPCI	cardiac death, non-fatal MI, unstable angina and new-CHF	No	1	NR	Fair
Dong PS	2011	Mainland China	Prospective	STEMI,PPCI	death, reinfarction, TLR, and new-CHF	Yes	1	ZEEK	Fair
Guo YS	2011	Mainland China	Prospective	STEMI,PPCI	Cardiac death, reinfarction and acute HF	Yes	In hospital	GOODMAN	Fair
Huang S	2013	Mainland China	Prospective	STEMI,PPCI	mortality	Yes	In hospital	ZEEK	Fair
Huang WG	2010	Mainland China	Randomized	STEMI,PPCI	death, reinfarction, TLR,	Yes	In hospital	ZEEK	Fair
Jia XG	2009	Mainland China	Prospective	STEMI,PPCI	death, reinfarction, subacute stent thrombosis	Yes	In hospital	ZEEK	Fair
Zhou DH	2012	Mainland China	Randomized	STEMI,PPCI	NR	No	3	Driver C.E.	Fair
Liu W	2012	Mainland China	Prospective	STEMI,PPCI	NR	No	In hospital	Driver C.E.	Fair
Pan G	2011	Mainland China	Randomized	STEMI,PPCI	death, reinfarction, and new-CHF	Yes	In hospital	Driver C.E.	Fair
Wang XM	2012	Mainland China	Randomized	STEMI,PPCI	death, reinfarction, TLR,	Yes	1	NR	Fair
Yang WM	2014	Mainland China	Randomized	STEMI,PPCI	death, reinfarction, TLR, and new-CHF	Yes	In hospital	EXPORT	Fair

PCI: percutaneous coronary intervention; PPCI: primary PCI; STEMI: ST-elevation myocardial infarction; MACE: major adverse cardiac events; TA: thrombus aspiration; CHF: congestive heart failure; TLR: target lesion revascularization; NR: not reported.

**Table 2:** Baseline and procedural characteristics in 7 reported studies.

Characteristics	Liu XY(2013)		Liu CP(2013)		Chen CW(2013)		Huang S(2013)		Huang WG(2010)		Jia XG(2009)		Wang XM(2012)	
	G_TA_(n=40)	G_PCI_(n=40)	G_TA_(n=28)	G_PCI_(n=23)	G_TA_(n=44)	G_PCI_(n=46)	G_TA_(n=31)	G_PCI_(n=96)	G_TA_(n=42)	G_PCI_(n=42)	G_TA_(n=36)	G_PCI_(n=32)	G_TA_(n=32)	G_PCI_(n=32)
Mean Age (years)	64.5	66.7	59	57	62	61	52.6	51.7	68.7	68.3	67.4	68.2	64.9	65.4

Male (n,% )	-	-	26 (92.6%)	20 (87.0%)	26(59.1%)	28(60.7%)	25(80.6%)	78(81.3%)	31(73.8%)	29(69.5%)	25(69.4%)	22(68.8%)	23(72%)	25(78.%)

Hypertension (n,% )	-	-	13 (46.4%)	13 (56.5%)	23(52.3%)	25(54.3%)	20(64.5%)	59(61.5%)	32(76.2%)	30(71.4%)	18(50.0%)	17(53.1%)	22(69%)	20(63%)

DM (n,% )	-	-	7(25.0%)	5(21.7%)	18(40.9%)	18(39.1%)	6(19.4%)	17(17.7%)	20(47.6%)	22(52.4%)	20(55.6%)	18(56.3%)	6(19%)	5(16%)

Hypercholesterolemia (n,% )	-	-	9(32.1%)	7(30.4%)	20(45.5%)	24(52.2%)	17(54.8%)	51(53.3%)	-	-	8(22.2%)	6(18.8%)	14(44%)	10(31%)

Current smoker (n,% )	-	-	16(57.1%)	13(56.5%)	17(38.6%)	18(39.1%)	21(67.7%)	62(64.5%)	-	-	15(41.7%)	13(40.6%)	20(63%)	16(50%)

Infarct location, anterior/inferior and posterior wall (n)	23/17	24/16	11/17	12/11	22/22	24/22	-	-	20/22	18/24	10/26	9/23	-	-

Postoperative TIMI grade 3 flow (n,% )	36(90.0%)	33(82.5%)	27(96.4%)	18(78.3%)	41(93.2%)	36(78.3%)	29(93.5%)	65(64.6%)	39(92.9%)	31(73.8%)	33(91.7%)	25(78.1%)	30(94%)	23(72%)

Postoperative LVEF in hospital or follow-up (%)	53.6	45.6	57	53	51.2	48.25	56.7	55.8	58.2	46.8	55.2	50.2	59.3	45.8

G_TA_: the group for intracoronary glycoprotein IIb/IIIa inhibitors after thrombus aspiration; G_PCI_: the group for percutaneous coronary interventions alone; DM: diabetes mellitus; LVEF: left ventricular ejection fraction; TIMI: thrombolysis in myocardial infarction trial.

**Table 3:** Subgroup meta-analysis of postoperative TIMI grade 3 flow and MACE according to TA catheter.

TA catheter	Number of studies	Postoperative TIMI grade 3 flow		MACE	
		OR (95% CI)	P value	OR (95% CI)	P value
ZEEK	5	2.52 (1.01, 6.31)	P=0.05	0.22 (0.14, 0.35)	P<0.00001

Driver C.E.	3	4.97 (2.03, 12.15)	P=0.0004	0.22(0.02, 2.14)	P=0.19

EXPORT	2	8.75 (2.92, 26.26)	P=0.0001	0.38 (0.14, 1.0)	P=0.05

TA: thrombus aspiration; OR: odds ratio; 95% CI: 95% confidence interval; TIMI: thrombolysis in myocardial infarction trial; MACE: major adverse cardiac events.

## Data Availability

The datasets generated and/or analyzed during the current study are available from the corresponding author on reasonable request.
